# Immune Checkpoint Inhibitor Therapy for Prostate Cancer: Present and Future Prospectives

**DOI:** 10.3390/biom15060751

**Published:** 2025-05-22

**Authors:** Vikrant Rai

**Affiliations:** Department of Translational Research, Western University of Health Sciences, Pomona, CA 91762, USA; vrai@westernu.edu; Tel.: +1-909469-7043

**Keywords:** prostate cancer, molecular mechanisms, immunotherapy, combination therapies, limitation to immunotherapy

## Abstract

Prostate cancer, a slow-growing tumor, develops through the over-proliferation of malignant cells in the prostate and is one of the most common types of cancer. Active surveillance, radical prostatectomy, external beam radiation, brachytherapy, cryotherapy, stereotactic body radiation therapy, hormone therapy, and chemotherapy are common treatment strategies for prostate cancer. However, resistance to treatment in advanced prostate cancer is a concerning issue in the use of these therapies. Immune checkpoint inhibitor (ICI) therapy for prostate cancer is an emerging strategy for the treatment of advanced prostate cancers but the resistance and limited efficacy to ICIs observed in metastatic castration-resistant prostate cancer (mCRPC) raises concerns. The ongoing clinical trials for combination therapies for mCRPC have provided some hope. This review concisely discusses the molecular and cellular mechanisms, immunotherapy, the limitations of ICIs, combination therapies, and the prospects of developing novel therapeutics for prostate cancer.

## 1. Introduction

According to the American Cancer Society, about 42 out of 100 men living in the United States will develop cancer at some point during their lifetime, and prostate cancer (PCa) is the most common cancer diagnosed in men, with one in eight men expected to develop PCa during their lifetime. The American Urological Association (AUA) reported approximately 288,300 newly diagnosed cases of PCa and 34,700 deaths related to PCa in the U.S. in 2023 [1]. As per the European Urology Association (EUA), the estimated age-standardized rate (ASR) of PCa was 31 per 100,000 males and the lifetime cumulative risk was 3.9%, with the highest all-age incidence ASR in Northern Europe (83 per 100,000), followed by Western Europe. In Europe, the all-age incidence ASR was 63 per 100,000 males in the year 2020, the lifetime cumulative risk was 16%, and all-age mortality ASR was 11 per 100,000 males varying in different regions of Europe [2]. PCa, which usually grows very slowly, is the fifth leading cause of overall death worldwide and the second leading cause of cancer-related death among men in the United States [3]. Aging is strongly related to the incidence and mortality rates of PCa, with >65 years of age associated with the highest incidence. African American men have more aggressive tumors and higher incidence compared to white men. In addition to aging, a diet rich in saturated animal fat and red meat, lower intake of fruits, vegetables, vitamins, and coffee, hyperglycemia, obesity, physical inactivity, chronic inflammation, infections, family history, endogenous hormones, and exposure to chemicals (insecticides, herbicides, and other organic compounds) or ionizing radiation are common risk factors for PCa [3,4]. A retrospective analysis including 955 patients (395 with PCa and 238 with advanced tumor) suggested metabolic syndrome and physical inactivity as risk factors for PCa. The study concluded that metabolic syndrome is an independent predictor of high-grade PCa [5]. However, the exact cause of PCa is largely unknown. The early stages of PCa are asymptomatic, while difficulty urinating, frequent urination, blood in the urine or semen, pain or discomfort during urination, and erectile dysfunction are common symptoms when they do occur at later stages. Digital rectal exam, prostate-specific antigen, and biopsy are the methods of diagnosis, while active surveillance (for less aggressive tumors), radical prostatectomy, external beam radiation, brachytherapy, cryotherapy, stereotactic body radiation therapy, hormone therapy, and chemotherapy are common treatment options for PCa [6,7].

Targeted therapies, including hormonal treatment, chemotherapy, radiotherapy, and immunotherapy, are used in advanced and metastatic prostate cancers. The use of hormone therapy and radiotherapy in prostate cancer treatment is associated with side effects including erectile dysfunction, urinary incontinence, decreased libido, hot flashes, bone loss, muscle loss, blood lipid changes, insulin resistance, and bowel problems [8]. Targeting prostate-specific membrane antigen (PSMA), a transmembrane glycoprotein that is specific and highly expressed in PCa, has shown significant progress in the last two decades. Radioligand therapy (^177^Lu-PSMA-RLT, ^225^Ac-PSMA-RLT), antibody–drug conjugates (MLN2704, PSMA-MMAE, MEDI3726), cellular immunotherapy (CAR-T, CAR/NK-92, PSMA-targeted BiTE), PSMA-targeted photodynamic therapy, PSMA-PET, PSMA-targeted RGS, and multimodal PSMA-targeted imaging guided therapy are common PSMA-targeted therapies with encouraging anti-tumor activity in PCa [9]. However, the side effects associated with PSMA targeted therapy warrant further research and clinical trials. Gonadotropin-releasing hormone (GnRH) agonists (Degarelix) and GnRH antagonists (buserelin, goserelin, leuprorelin, and triptorelin) are initial hormonal therapies for PCa but these are associated with an increased risk of adverse cardiovascular events. Cicione et al. [10] reported a higher association of cardiovascular events, including hypertension, thrombosis, and myocardial infarction, with GnRH antagonists compared to GnRH agonists. Thus, these drugs should be prescribed with precaution in PCa patients with cardiovascular complications.

Newer therapies, including high-intensity focused ultrasound, docetaxel, cabazetaxel (for advanced hormone-refractory prostate cancer), abiraterone (an oral androgen biosynthesis inhibitor for the treatment of advanced metastatic castrate-resistant prostate cancer), denosumab (a monoclonal antibody), olaparib (PARP (poly(ADP-ribose) polymerase) inhibitor; targeted therapy), and sipuleucel-T (a new vaccine; immunotherapy) are emerging therapies for metastatic and resistant tumors [6,7]. The randomized, double-blind, phase III KEYNOTE-921 trial reported no significantly improved efficacy after the addition of pembrolizumab to docetaxel for the treatment of metastatic castration-resistant prostate cancer (mCRPC) [11]. A recent study reported a long-term process for the recovery of total testosterone levels after androgen-deprivation therapy cessation [12]. The therapeutic response of systemic therapy in PCa is usually transient, and invasive disease is associated with high mortality rates. This suggests that even the newer therapies are associated with side effects or are not sufficient for refractory PCa or the recurrence of PCa, and there is a need for a more robust therapeutic strategy for treatment-resistant advanced PCa. The emergence of immunotherapy as an efficacious and non-toxic alternative treatment is because of its immune-targeted molecular traits, which can achieve the maximum clinical benefit. This review summarizes the recent developments in immunotherapy for PCa.

## 2. Molecular Mechanisms of PCa Progression and Immunosuppression

**Figure 1:**
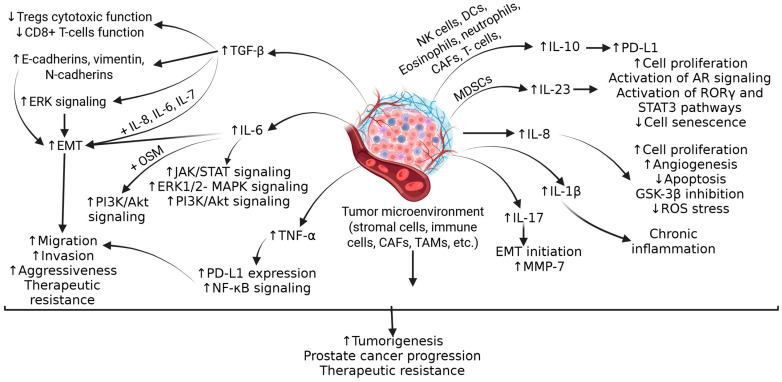
The tumor microenvironment (TME) induces and contributes to an immunosuppressive environment in advanced prostate cancer. The cytokines, growth factors, and activation of downstream signaling pathways contribute to increased tumorigenesis, tumor progression, and therapeutic resistance. IL—interleukin; Tregs—T regulatory cells; ERK—extracellular signal-regulated kinase; EMT-epithelial–mesenchymal transition; OSM—oncostatin-M; PI3K—phosphoinositide 3-kinase; Akt—also known as protein kinase B; MAPK-mitogen activated protein kinase; JAK/STAT—Janus kinase, a signal transducer and activator of transcription; TGF-β—transforming growth factor beta; TNF-α—tumor necrosis factor alpha; PD-L1—programmed cell death ligand 1; NF-kB- nuclear factor kappa beta; CAFs—carcinoma-associated fibroblasts; TAMs—tumor associated macrophages; MDSCs—myeloid-derived stem cells; NK cells—natural killer cells; DC—dendritic cells; AR—androgen receptor; MMP—matrix metalloproteinase; ROS—reactive oxygen species; ROR γ—RAR-related orphan receptor gamma.”↑”—increased expression; “↓”—decreased expression.

Understanding the molecular mechanisms underlying PCa is important because immunotherapy uses the body’s own immune system to fight cancer by stimulating or enhancing the immune system’s ability to recognize and destroy cancer cells. Inflammation and immune cells play a critical role in the pathogenesis of PCa. The role of CD3+, CD8+, and CD4+ T cells; B-cells; tumor-associated macrophage (TAM); dendritic cells (altered function due to the increased secretion of transforming growth factor (TGF)-β and interleukin (IL)-10)); immunosuppressive immune cells including T regulator cells (Treg), myeloid-derived suppressor cells (MDSC), Th17 cells, macrophages, mast cells, and neutrophils involving IL-2; interferon (IFN)-γ; IL-1β; IL-1α; IL-1RA; IKKα, a signal transducer and activator of transcription 3 (STAT3); BMI1, C-X-C chemokine ligand (CXCL)13; and C-C chemokine ligand (CCL)2 in the pathogenesis, progression, and aggressiveness of PCa has been discussed [13]. Tumor cells, stromal cells, and other immune cells in the tumor microenvironment (TME) secrete TGF-β, contributing to the attenuated function of Tregs, CD8+ T-cells, which activate ERK and promote epithelial–mesenchymal transition (EMT), leading to the growth and increased invasiveness of PCa (Figure 1).

**Figure 2:**
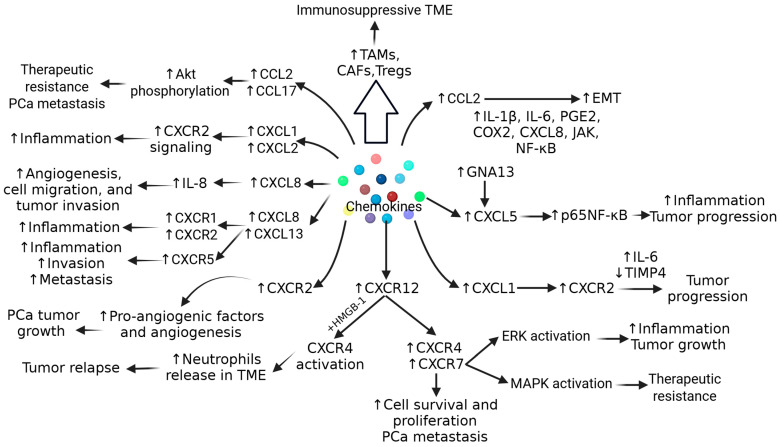
Role of chemokines in progression, tumorigenesis, and therapeutic resistance in prostate cancer (PCa). IL—interleukin; Tregs—T regulatory cells; ERK—extracellular signal-regulated kinase; EMT—epithelial–mesenchymal transition; Akt—also known as protein kinase B; MAPK—mitogen activated protein kinase; JAK—Janus kinase; NF-kB—nuclear factor kappa beta; CAFs—carcinoma associated fibroblasts; TAMs—tumor-associated macrophages; TIMP—tissue inhibitor of matrix metalloproteinase; TME—tumor microenvironment; CXCL—C-X-C motif ligand; CXCR—C-X-C motif receptor; CCR—C-C motif chemokine receptor; CCL—C-C motif chemokine ligand; COX2—cyclooxygenase 2; PGE2—prostaglandin E2; HMGB1—high-mobility group box protein 1. “↑”—increased expression; “↓”—decreased expression.

IL-6 secreted by TME cells activates Janus kinase-signal transducer and activator of transcription (JAK/STAT), extracellular signal-regulated kinase (ERK)1/2-mitogen activated protein kinase (MAPK), and phosphoinositide 3-kinase (PI3K) pathways, and contributes to cell proliferation, EMT, tumor migration, increasing its growth and aggressiveness, and resistance to chemotherapy by inducing anti-apoptotic pathways. (Figure 1). IL-10, an anti-inflammatory cytokine secreted by various immune cells in TME cells, is correlated with poor prognosis in PCa and contributes to immunosuppression via increased expression of PD-L1. Another cytokine, IL-23, secreted by MDSCs, activates STAT3, RAR-related orphan receptor gamma (RORγ), and androgen receptor signaling, leading to cell proliferation, CRPC growth and metastasis, and treatment resistance to anti-androgen therapy (Figure 1). Cytokines such as IL-8, IL-1β, IL-17, IFN-γ, and tumor necrosis factor (TNF)-α play a critical role in cell proliferation, chronic inflammation, modulating apoptosis, angiogenesis, and the progression of PCa (Figure 1). It should be noted that the increased expression of TGF-β, IL-6, IL-10, and IL-23 is associated with poor prognosis in PCa [14,15]. Furthermore, along with these cytokines, CC and C-X-C chemokines including CCL2, CCL22, CCR4, CCL17, CXCL2, CXCR2, CXCL8, CXCL13, CXCL5, CXCR1, CXCR2, CXCL1, CXCL2, CXCL12, and CXCR4 play a critical role in pathogenesis, tumor heterogeneity, and immunosuppressive TME in PCa (Figure 2) [15].

**Figure 3:**
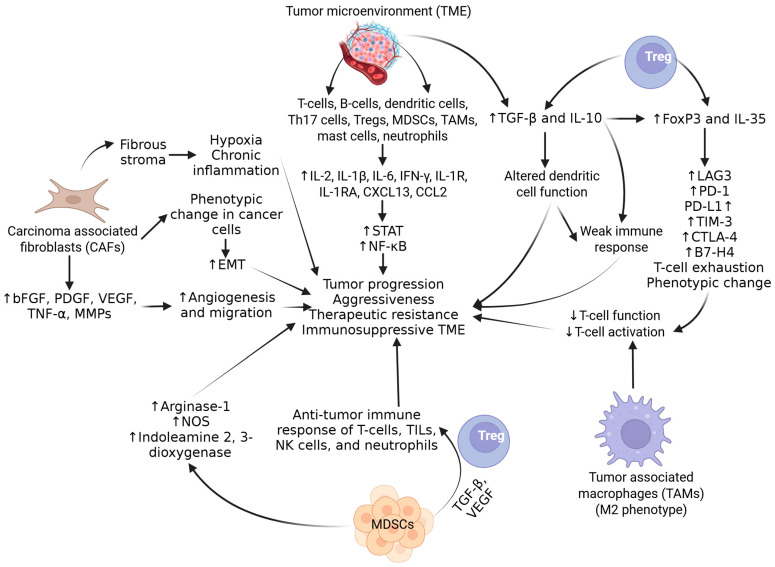
Role of tumor microenvironment components in inducing immunosuppression and therapeutic resistance. IL—interleukin; Tregs—T regulatory cells; EMT—epithelial–mesenchymal transition; STAT—signal transducer and activator of transcription; TGF-β—transforming growth factor beta; TNF-α—tumor necrosis factor alpha; PD-1—programmed cell death protein 1; PD-L1—programmed cell death ligand 1; NF-kB—nuclear factor kappa beta; CAFs—carcinoma-associated fibroblasts; TAMs—tumor-associated macrophages; MDSCs—myeloid-derived stem cells; NK cells—natural killer cells; MMPs—matrix metalloproteinases; IFN γ—interferon-gamma; TME—tumor microenvironment; CXCL—C-X-C motif ligand; CCR—C-C motif chemokine receptor; TILs—tumor-infiltrating lymphocytes; NOS—nitric oxide synthetase; VEGF—vascular endothelial growth factor; PDGF—platelet-derived growth factor; bFGF—basic fibroblast growth factor; LAG3—lymphocyte activation gene-3; TIM3—T-cell immunoglobulin and mucin domain 3; CTLA-4—cytotoxic T-lymphocyte-associated protein 4. ”↑”—increased expression; “↓”—decreased expression.

In addition to immune cells, TAMs, MDSCs, and carcinoma-associated fibroblasts (CAFs) present in the tumor microenvironment (TME) also contribute to tumor progression and aggressiveness. Fibroblasts secrete basic fibroblast growth factor (bFGF), platelet-derived growth factor (PDGF), vascular endothelium growth factor (VEGF), TNF-α, and MMPs, increasing migration and angiogenesis in tumors [16]. CAFs are also associated with a phenotypic change in cancer cells, causing them to act as stem cells undergoing epithelial–mesenchymal transition (EMT). CAF also contributes to fibrous stroma formation, causing hypoxia and chronic inflammation in the tumor microenvironment. Activated Tregs contribute to immunosuppressive TME and its maintenance by attenuating the antitumor immune response mediated by T cells, tumor-infiltrating lymphocytes, natural killer (NK) cells, and neutrophils. MDSCs, after binding to CXCL5 and IL-8-mediated chemotaxis, are recruited to the TME and contribute to immunosuppression via the activation of arginase 1, nitrous oxide synthase, and indoleamine 2,3-dioxygenase. The predominance of M2-like macrophages in TME reduces the T-cell function and contributes to tumor progression and immunosuppression (Figure 3) [17]. The immunosuppressive environment and attenuation of antitumor immune response can also be mediated through the epithelial immune-like transition of cancer cells mediated by various cytokines, receptors, Ig motifs, transcription factors, and immune checkpoint molecules, including PD-1, PD-L1 (B7-H3, modulating T lymphocyte activity), B7-H4 (expressed on activated T-cells, B-cells, dendritic cells, and monocytes, modulating the inhibition of T cells’ proliferation and cytokine production), T-cell immunoglobulin 3 (TIM-3, negatively regulating Th1 cell activity), lymphocyte-activation gene 3 (LAG3, binds to HLA class II and inhibits T-cell function), and cytotoxic T-lymphocyte-associated protein 4 (CTLA-4), restricting early T-cell activation [18] (Figure 3). Tregs play a critical role in the pathogenesis of PCa and are an attractive therapeutic target. Tregs suppress the function of naive T cells, B and T lymphocytes, dendritic cells, NK cells, and macrophages. The inhibition of immune response via Tregs is mediated through modulating and regulating cytokine release, apoptosis, antigen-presenting cells (APCs), and ATP degradation [19]. Tregs contribute to the growth, migration, and invasion of PCa via the increased secretion of IL-10, TGF-β, and IL-35, leading to the suppression of immune response, T cells’ exhaustion, and the increased expression of neoantigens such as lymphocyte activation gene-3 (LAG3), T-cell immunoglobulin and mucin domain 3 (TIM3), PD-1, and PD-L1 (Figure 3).

**Figure 4:**
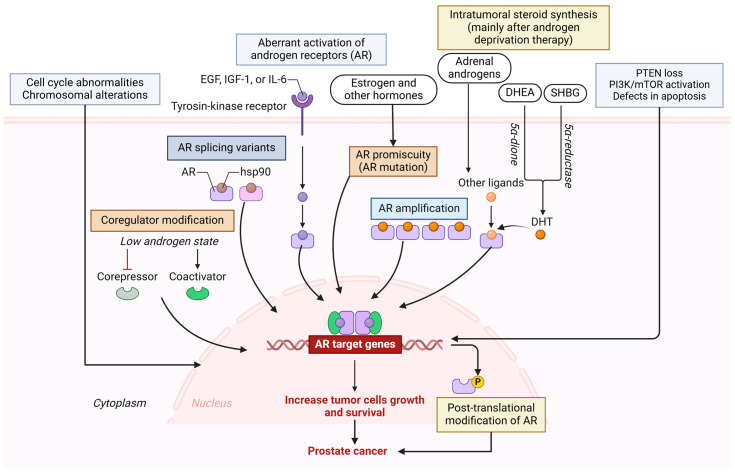
Molecular mechanisms involved in pathogenesis and resistance to therapy in advanced PCa. Multiple mechanisms include aberrant androgen receptor (AR) expression, AR mutation, AR splice variants, AR amplification, cell cycle abnormalities, defective apoptosis, loss of PTEN, activation of PI3K/mTOR pathways, and other factors that increase the growth and survival of tumor cells and contribute to pathogenesis and resistance to treatment in advanced PCa. PTEN—phosphatase and tensin homolog; PI3K—phosphoinositide 3-Kinase; mTOR—mammalian target of rapamycin; HSP90—heat shock protein 90; EGF—epidermal growth factor; IL6—interleukin 6; IGF1—insulin-like growth factor 1; DHEA—dehydroepiandrosterone; SHBG—sex hormone binding globulin; DHT—dihydrotestosterone. Created in BioRender. Vikrant Rai. (2025) https://app.biorender.com/.

DNA mutations cause abnormal cell growth and affect androgen receptor (AR) pathways, DNA repair pathways, and other pathways underlying PCa. Mutations, amplifications, and splicing variants in androgen receptor-related genes in AR pathways are common mechanisms underlying PCa. Failures in DNA repair can lead to genomic instability and tumor onset. Abnormalities in the cell cycle and PI3K pathway are associated with the onset and progression of PCa. Chromosomal alterations and rearrangements, the loss of phosphatase and tensin homolog (PTEN) and activation of phosphoinositide 3-Kinase/-mammalian target of rapamycin (PI3K/mTOR), the increased expression of interleukins and growth factors, global defects in apoptosis, and the loss of function of the prostate tumor-suppressor gene *NKX3* are other molecular aberrations associated with PCa [20,21,22,23,24] (Figure 4). Co-regulator modification (e.g., GRHL2) [25], co-transcriptional R-loops-mediated epigenetic regulation [26], and intratumoral steroid synthesis, mainly androgens, after androgen deprivation therapy play a critical role in metastatic castration-resistant PCa (mCRPC) progression [27], and androgen promiscuity, the situation in which mutated androgen receptors may be activated by alternative ligands, including estrogen and other hormones [28,29,30], which contribute to the progression and pathogenesis of PCa (Figure 4). These mechanisms also contribute to resistance to treatment. Chromosomal abnormalities, including deletion, addition, and amplification, are frequently found in PCa. Further, a decreased copy number and loss of heterozygosity in chromosome 8p are also associated with PCa [31]. Telomere attrition also plays a role in the pathogenesis, greater genotype and phenotype heterogeneity, aggressiveness, decreased overall survival, increased biochemical recurrence, and increased risk of death in PCa [32,33].

The role of TMPRSS2-ERG gene fusion, PTEN deletion, and MYC loss in the pathogenesis of PCa has been discussed. SLC45A3-ERG rearrangements co-occurring with TMPRSS2-ERG and in association with PTEN loss, the mutations in TP53 pathways, the mutation of the *ORZAP1* gene, mismatch repair gene alteration at the level of MSH2 or MSH6 genes, germline DNA repair gene mutations (*BRCA2*, *ATM*, *CHEK2*, and *BRCA1*), *CTNNB1* hot spot mutations, germline BRCA2 mutations, the genomic and epigenomic dysregulation of MED12L/MED12 axis, point mutations of *TP53*, *SPOP*, and *FOXA1*, the increased frequency of SPOP and ATM mutations, the loss of the retinoblastoma (RB) tumor-suppressor, the inactivation of *LRF* proto-oncogene, *CDK12* deletion, and mutations in *IDH1*, *MAP3K7*, and *CDH1* genes in the pathogenesis of PCa have been discussed [20,23]. Further, the rearrangement of MAG12 (PTEN inhibitor), BRAF, RAF, and CADM2, their overexpression and mutations in PIK3CA1, the deletion and downregulation of PHLPP1/2, a point mutation in Akt1, mutations in SPOP, CHD1, MLL2, ASH2L, and MED12, the overexpression of SPINK1, MYC, and NMYC, the elevated expression of EZH2 and BM1, the deletion of TAK1/MAP3K7, and the loss of TP53 also play a role in PCa pathogenesis [23].

Epigenetics plays a critical role in the pathogenesis of PCa. Dietary carcinogens (heterocyclic aromatic amines and polycyclic aromatic hydrocarbon due to red meat consumption), estrogens, and oxidants acting as a trigger for chronic inflammation promote PCa [24]. Hypermethylation in the promoter regions of tumor-suppressor genes leads to their inactivation in the pathogenesis of PCa. The most frequent somatic alteration in PCa is the aberrant methylation of the CpG island at the glutathione S transferase pi (GSTP1) locus in the presence of oxidative stress. However, this may also be positive in hyperplastic and normal tissue. GSTP1 polymorphisms have been associated with the risk of biochemical recurrence of PCa. Further, methylation of the Ras-association domain of the familial protein 1 isoform A (RASSF1A) located on chromosome 3p21 has also been reported [24]. Methylation has been reported in other genes, including androgen receptor, estrogen receptor 1 and 2, retinoic acid receptor β2, retinoic acid receptor responder 1, cyclin d2, cyclin-dependent kinase inhibitor 2a (p16), reprimo, stratifin (14-3-3 sigma), dickkopf 3, endothelin receptor type b, ras-association domain family protein 1 isoform a, runt-related transcription factor 3, secreted frizzled-related protein 1, familial adenomatous polyposis, caveolin 1, E-cadherin, cadherin 13, cluster-differentiation antigen 44, α-3 laminin, γ-3 laminin, TIMP metallopeptidase inhibitor 3, glutathione s-transferase m1, glutathione s-transferase p1, glutathione peroxidase 3, o-6-methylguanine DNA methyltransferase, apoptosis-associated speck-like protein containing a CARD, B cell lymphoma 2, death-associated kinase, multidrug-resistance receptor 1, and prostaglandin endoperoxidase synthase 2. Hypermethylation in genes involved in hormonal response, cell cycle control, signal transduction, tumor invasion, DNA damage repair, and apoptosis has also reported in PCa [34,35].

In addition to hypermethylation, post-transcriptional histone modifications involving histone deacetylases (HDACs, e.g., HDAC1), histone methyltransferases (HMTs, e.g., EZH2), and histone demethylases (HDMs, e.g., LSD1) also play a critical role in PCa. The enhancer of the zeste homolog 2 gene (EZH2) catalyzes the trimethylation of histone H3K27 and dimethylates H3K9, and its overexpression correlates with promoter hypermethylation and tumor aggressiveness. HDAC1 overexpression is associated with PCas containing TMPRSS2–ERG fusion. Lysine-specific demethylase 1 (LSD1) removes mono- or dimethyl groups from H3K4, acting as a transcriptional corepressor in PCa. Histone acetylation regulates androgen receptor regulation, and its activity is downregulated by HDAC1, HDAC2, HDAC3, and siRT1 [34]. The role of miRNAs, including miR-221/miR-222, miR-616, miR-125b, miR-488, miR-146a, miR-331-3p, miR-21, miR-34c, miR-34a, miR-15a, miR-16-1, miR-125b, miR-145, let-7c, and others, has also been discussed in the literature [34,35].

## 3. Immunotherapy for PCa

Immunotherapy is an emerging strategy for the treatment of advanced PCas that are resistant to other treatments, such as hormone therapy and chemotherapy. PCa showing a high tumor mutational burden, high programmed cell death protein 1 (PD-L1) tumor expression, cyclin-dependent kinase 12 (CDK12) mutations, mismatch repair-deficient (dMMR), a high microsatellite instability, homologous recombination deficiency, and (BRCA2 and ATM) POLE/POLD1 mutations, as well as patients in a good status of health, provide ideal candidates for immunotherapy because these changes increase response to immunotherapy by increasing the tumor mutation burden and neoantigen expression [36,37,38]. Immunotherapy uses patients’ own immune system to fight cancer cells while sparing healthy cells. Immunotherapy is effective because of its ability to recognize and eliminate newly developing cancer cells. Immunotherapy may also augment the therapeutic efficacy of other treatments when used in combination. The two main types of immunotherapies used for PCa are cancer vaccines targeting tumor-specific antigens and using monoclonal antibodies. Cancer vaccines stimulate the immune system to recognize and attack PCa cells and sipuleucel-T (Provenge, a dendritic cell vaccine pulsed with a chimeric protein expressing GM-CSF and PAP as a tumor-associated antigen) is the most common vaccine used for PCa. Immune checkpoint inhibitors (ICI) block proteins in the cancer cells that help them evade the immune system and pembrolizumab (Keytruda) and nivolumab (Opdivo) are the most common ICIs used for PCa. It should be noted that immunotherapy is not a cure for PCa and may not be effective for all patients [39,40].

Antibody–drug conjugates, artificial bi-specific T cell-engaging antibodies, and immune checkpoint inhibitors are approaches to use monoclonal antibodies, while chimeric antigen receptor (CAR)-T cell therapy is a strategy for the adoptive transfer of T cells; both are part of immunotherapy [39] (preclinical and phase I clinical trial). Immune checkpoint inhibitors are monoclonal antibodies that inhibit immune checkpoint receptors to prevent the inactivation of T-cell function, and these antibodies target receptors, including cytotoxic T lymphocyte-associated protein 4 (CTLA-4), programmed death 1 (PD-1), and programmed death ligand 1 (PD-L1) [39].

### 3.1. Cytotoxic T Lymphocyte-Associated Protein 4

CTLA-4, a transmembrane protein expressed on T lymphocytes, competitively binds to ligands CD80 (B7.1) and CD86 (B7.2) on antigen-presenting cells (APCs), and prevents T-cells from killing other cells, including cancer cells [41]. Ipilimumab, a CTLA-4 inhibitor and the first Food and Drug Administration (FDA)-approved immune checkpoint inhibitor (ICI), blocks CTLA-4, switches off the inhibitor mechanism, and potentiates T-cell effects. CTLA-4 monotherapy significantly increases the ratio of regulator effector T lymphocytes in the tumor microenvironment [42]. However, monotherapy was not found to be associated with increased overall survival and failed to achieve significant benefits [43]. Ipilimumab showed promising results of a significant decline in prostate-specific antigen (PSA) levels when given in combination with granulocyte macrophage colony-stimulating factor (GM-CSF) and anti-tumor activity in phase I and phase I/II clinical trials, respectively, in metastatic castration-resistant PCa (mCRPC). A phase III clinical trial showed significantly increased overall survival (OS) in mCRPC but no significant change in OS was reported in asymptomatic or minimally symptomatic chemotherapy-naïve PCa with Ipilimumab monotherapy [38,39]. A study by Witt et al., using a mouse model of PCa, reported that signal transducer and activator of transcription 3 (STAT3) inhibition by GPB730 enhances the antitumor efficacy and activity of anti-CTLA-4, along with decreasing intratumoral Treg frequency, supporting the combination of STAT3 inhibition with anti-CTLA-4 therapy [44].

### 3.2. Programmed Death 1

PD-1 expressed on activated T cells, B-cells, and natural killer (NK) cells, when bound with PD-L1 (expressed on both normal and malignant cells), inhibits T cell activation (checkpoint for T lymphocyte inhibition) and converts naïve T cells into regulatory (Tregs) cells. Tumor cells expressing PD-L1 evade T cell antitumor response involving anergy or apoptosis of the effector T cells because the binding of PD-1 with PD-L1 results in apoptosis inhibition, T-lymphocyte tolerance, and increased tumor cell survival [45]. PD-L1 expression in PCa correlates with shorter clinical failure-free survival, proliferation (Ki-67), a lower Gleason score, and reduced prognostics for biochemical recurrence, but not with outcomes. Further, the expression profile in various studies also varied; these findings may be due to a lack of uniform criterion for determining PD-L1 positivity [38].

Nivolumab and pembrolizumab are ICIs for PD-1 inhibition, while atezolizumab, avelumab, and durvalumab are ICIs for PD-L1 inhibition [41]. Nivolumab or pembrolizumab have shown partial response in prostate tumors resistant to enzalutamide and with microsatellite instability (MSI). Patients with DNA mismatch repair mechanism (MRM) mutations may benefit from pembrolizumab [39]. Pembrolizumab, as a monotherapy or in combination (KEYNOTE-028 and KEYNOTE-199), induces durable objective responses, with minimal adverse events, and leads to anti-tumor activity with durable clinical responses and significant OS in patients with advanced PD-L1-positive mCRPC; therefore, it has been suggested as a form of immunotherapy. A high tumor mutational burden (TMB), PD-L1 expression, and high microsatellite instability (MSI) are the biomarkers for Pembrolizumab therapy [38,46,47,48]. Atezolizumab is a PD-L1 antibody, and its monotherapy has shown safety and clinical activity in terms of disease control and 12-month OS in mCRPC patients in a phase Ia, open-label, dose-escalation and dose-expansion study [49]. However, combination therapies are needed. The combination therapies of PD-L1 blockade with androgen inhibitors including The IMbassador 250 trial (atezolizumab with enzalutamide or enzalutamide alone) showed no overall significant improvement in OS [50] in KEYNOTE 365, COHORT C (enzalutamide in combination with pembrolizumab), showing a PSA response rate of 22% and ORR of 12% but not reaching the median duration of response [51], and in KEYNOTE 199 (enzalutamide plus pembrolizumab combination), showing a 12% ORR and 70–75% OS but not reaching median OS [52]. This suggests the need for more large-scale clinical trials with combination therapies.

### 3.3. Anti-Tumor Vaccines

The presence of tumor-associated antigens (TAA) in PCa makes it suitable for anti-tumor vaccines that elicit an adaptive immune response via antigen presentation [41]. Cell-based vaccines (dendritic cell or tumor cell), vector-based vaccines, DNA/mRNA-Based vaccines, and antigen- or peptide-based vaccines are common vaccines for PCa (Table 1). Vector-based vaccines include vectors derived from oncolytic viruses or bacterial pathogens inducing a specific immune response. DNA- and RNA-based vaccines consisting of plasmid DNA and mRNA, respectively, which elicit an immune response via both the major histocompatibility complex (MHC) class I and MHC class II pathways. The high potency, low manufacturing cost, developmental feasibility, and acceptable safety profile of DNA- and RNA-based vaccines make them an alternative to conventional vaccines [39,53].

Antigen or peptide-based vaccines leverage short chains of amino acids (peptides) mimicking or representing specific parts of the antigens (epitopes) that best stimulate the immune system, and thus offer a targeted approach for inducing protective or therapeutic responses against cancer. The peptides presented to the body’s immune system lead to the production of antibodies and the activation of T cells. These activated T cells and antibodies then recognize and destroy the target cells carrying the same antigen. High purity, a minimal risk of side effects, potential autoimmunity, the inclusion of multiple antigens or epitopes in a single vaccine, and the ability to tailor vaccines to specific tumor types or individual patient characteristics are advantages of antigen or peptide-based vaccines [54,55]. Using these vaccines constitutes an attractive immunotherapeutic approach called personalized peptide vaccination (PPV) [56]. PPV plus chemotherapy, PPV plus glucocorticoids, HER2/neu peptides, and the AE37 vaccine are the most promising immunotherapeutic platforms involving PPV, as discussed by Adamaki et al. [39].

## 4. Combination Therapies

In mCRPC, combination therapies have shown promising results (Table 2), and various clinical trials are ongoing (Tables 2 and 3 of [57]) to evaluate their efficacy and safety.

## 5. Therapies for Neuroendocrine Prostate Cancer and Castration-Resistant Prostate Cancer

Table 3 lists other therapies for neuroendocrine prostate cancer (NEPC) and castration-resistant prostate cancer (CRPC, in addition to Table 1 and Table 2). NEPC, a rare tumor, is a highly aggressive variant of CRPC. When adenocarcinoma is treated with hormonal therapies , it can transform to NEPC, with a poor prognosis. Currently, NEPC lacks specific treatment options, and the available treatments are ineffective. Potential novel targets for NEPC are zeste homolog 2 (EZH2) inhibitors, lysine-specific demethylase 1 (LSD1) inhibitors, poly-ADP-ribose polymerase (PARP) inhibitors, aurora kinase A inhibitors, and delta-like ligand 3 (DLL3)-targeted therapies [74]. A recent study suggested Lewis Y antigen as a novel target for CAR-T cell therapy in patients with NEPC [75].

Other therapies for neuroendocrine prostate cancer targeting SSTR include somatostatin receptor; DLL-3, delta-like ligand 3; GPC3, glypican-3; GRPR, gastrin-releasing peptide receptor; B7-H3; CXCR2, CXC chemokine receptor 2; NCAM1, neural cell adhesion molecule 1; CEACAM5, carcinoembryonic antigen-related cell adhesion molecule 5; and HEPACAM2, HEPACAM Family Member 2. These have been summarized elsewhere [80]. Additionally, various clinical trials, including NCT05652686 (PT217), NCT04702737 (AMG757), NCT03480646 (CPI-1205), NCT02875548 (Tazemetostat), NCT05293496 (Vobramitamab and Duocarmazine), NCT04926181 (Cetrelimab, JNJ-63723283), NCT04592237 (Cetrelimab, JNJ-63723283), and NCT03866382 (Cabozantinib), are exploring the role of various agents in neuroendocrine prostate cancer [81]. For NEPCs, ICIs alone have achieved limited success in NEPC; they are being explored in combination with other therapies. For example, a combination therapy with ADT and immune checkpoint inhibitors has been investigated. Immunotherapeutic approaches for NEPC are still in their early stages, but promising preclinical and clinical data suggest that targeted therapies, including DLL3-targeted ADCs and bispecific antibodies, have the potential to treat this aggressive form of prostate cancer. Further research is needed to optimize immunotherapy strategies and identify the best combination therapies for NEPC patients [81,82].

## 6. Limitations of Immunotherapy

The immunologically cold behavior, strong immunosuppressive tumor microenvironment, and low tumor mutation burden observed in PCa limit the use of immunotherapy [83]. As discussed above, TAMs, fibroblasts within the tumor stroma, Trges, and MDSCs are present in TME, and molecules contributing to immunosuppression and the increased expression and activation of TGF-β, IL-10, IL-6, IL-8, prostaglandin E2, VEGF, PD-1, and PD-L1 are secreted by these cells. These factors further contribute to resistance to immunotherapy [17]. The poor T cell infiltration (reason for poor response to immunotherapy) in PCa [84,85] is due to a hypoxic environment [86] causing an acidic pH [87], the depletion of essential nutrients, the increased expression of adenosine and PD-L1 [88], and the increased expression of immunosuppressive transforming growth factor (TFG)-β. Primary PCas are characterized by PD-1-expressing CD8+ T cell infiltration, but this decreases in mCRPC, and thus a poor immune response is shown for ICIs targeting PD-1/PD-L1. However, mCRPC that expresses PD-L1 and develops resistance to the anti-androgen enzalutamide responds to ICIs inhibiting PD-1/PD-L1 [38,39]. T cells in the tumor cells (CD4+ FOXP3+ CD25+ T cells and CD8+ FOXP3+ CD25+ T cells) are immunosuppressive due to their increased secretion of inhibitory cytokines and inhibition of naïve T cell proliferation. The decreased expression of major histocompatibility complex (MHC) class I, low tumor mutation burden, low PD-1 expression, chronic activation of the interferon-1 (IFN-1), and PTEN loss adversely affect the tumor microenvironment and immunotherapy outcomes [38,41,83]. The exhaustion or dysfunction of infiltrating T cells, rendering them unable to effectively target and kill cancer cells, also contributes to their nonresponsiveness to ICIs [89]. The presence of myeloid-derived suppressor cells (MDSC) in the tumor microenvironment suppresses cytotoxic T-cell infiltration and restricts the anti-tumor effects of immune checkpoint inhibitors [43].

Tumor heterogeneity adds more complexity to the treatment of PCa because tumors expressing PD-L1 and not responding to immunotherapy are due to tumor heterogeneity. PD-L1 expression also varies with tumor progression and its expression is modulated by chemotherapy and radiation therapy [38]. Further, the presence of cancer-associated fibroblasts (CAFs) regulating tumor microenvironment modifies the response to immune checkpoint inhibitors and contributes to chemoresistance [90,91]. A tumor-immune microenvironment with a low number of tumor-infiltrating lymphocytes (TILs) and a high number of tumor-associated macrophages (TAMs) in PCa is unique and resistant to ICIs [92]. Racial difference has also been suggested as a factor for the different outcomes obtained in the treatment of PCa due to differential immune responses [40]. A lower TMB associated with fewer neoantigens hindering T-cell priming [93,94], an impaired T-cell priming and activation due to defects in tumor antigen presentation [94], the inhibitory effect of androgen deprivation therapy on the immune system [95], and the lack of effective biomarkers potentially reduce the effectiveness of ICI therapy. The use of a combination of ICIs is a current area of research aiming to overcome these limitations (Table 1 of [39] and Table 2 of [38]).

## 7. Future Prospectives

**Figure 5:**
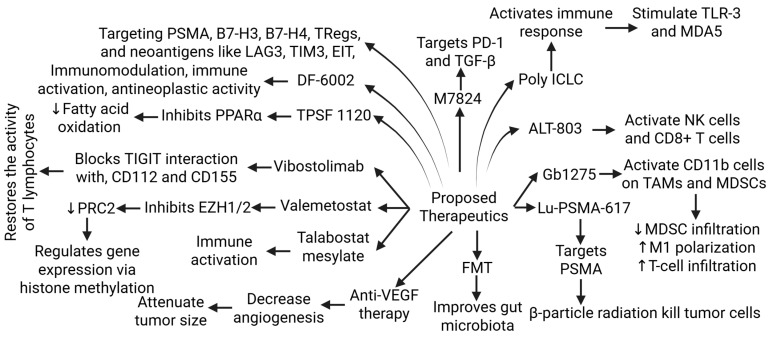
Proposed newer therapies for metastatic chemoresistant prostate cancer (mCRPC) undergoing pre-clinical or clinical trials. Tumor-associated macrophages (TAMs), myeloid-derived stem cells (MDSCs), toll-like receptor (TLR)-3, peroxisome proliferator-activated receptor alpha (PPARα), polycomb repressive complex 2 (PRC2), T cell immunoreceptor with Ig and ITIM domains (TIGIT), enhancer of zeste homolog (EZH) 1, prostate-specific membrane antigen (PSMA), lymphocyte activation gene 3 (LAG3), T-regulatory cells (Tregs), T-cell immunoglobulin and mucin-domain containing-3 (TIM-3), epitope-based immunotherapy (EIT), Programmed Cell Death 1 (PD-1), transforming growth factor (TGF)-β, melanoma differentiation-associated protein (MDA) 5, natural killer (NK) cells, fecal microbial transplantation (FMT), and vascular endothelial growth factor (VEGF). ”↑”—increased expression; “↓”—decreased expression.

The inconsistent and ineffective results of CTLA-4 and PD-1/PD-L1 monotherapy and the ongoing trials of combination therapies suggest the need for further research and novel strategies for the treatment of mCRPA. As discussed above, a “cold” tumor type, limited ICI efficacy, the tumor microenvironment, the presence of CAFs, MDSC, and TAMs, and the lack of specific ICI therapy approval from the FDA are current challenges in the treatment of mCRPC. Combination immune checkpoint and adenosine axis blockade, Bispecific T cell engager and immune checkpoint blockade, Lu-PSMA-617 and immune checkpoint blockade, adoptive T-cell therapy, and immune checkpoint blockade [96,97,98] have been suggested as potential therapies to improve clinical outcome [38] (Figure 5). Research and clinical trials related to other strategies, such as fecal microbiota transplant (to improve gut microbiota), VEGF receptor-inhibitors (to decrease neoangiogenesis, thereby decreasing tumor growth), TPST-1120 (a peroxisome proliferator-activated receptor alpha (PPARα) antagonist that reduces fatty acid oxidation and shifts tumor cells to a more glycolytic metabolism), valemetostat-EZH1/2 dual inhibitor (inhibits the polycomb repressive complex 2 (PRC2), regulating gene expression through histone methylation), DF6002 (a monovalent IL-12 immunoglobulin Fc fusion protein that is immunostimulant, immunomodulatory, and antineoplastic), and M7824 (a fusion protein with two extracellular domains of TGF-RII and a PD-L1 monoclonal antibody (targets PD-1 and TGF-β)) are ongoing to improve clinical outcome in resistant PCa (Figure 5).

Poly ICLC, a synthetic double-stranded RNA complex activating innate immune responses, primarily through the stimulation of TLR3 and MDA5 receptors; ALT-803, a recombinant IL15 complex that enhances anti-tumor immune responses by directly activating NK cells and CD8+ T cells; GB1275,—a CD11b modulator that binds to and activates CD11b on TAMs and MDSCs in TME, which leads to reduced MDSC infiltration, the repolarization of M2 TAMs to an M1 phenotype, and increased tumor infiltration of cytotoxic T cells; vibostolimab, a monoclonal antibody that binds to the T-cell immunoreceptor and blocks its interaction with its ligands (targets and blocks the TIGIT receptor, an immune checkpoint protein); and Talabostat Mesylate, a small molecule inhibitor of dipeptidyl peptidases (acts through dipeptidyl peptidase inhibition and immune system activation) are under clinical trials to potentiate the treatment of mCRPC (as discussed in Table 3 of [38]) (Figure 5).

Adenosine has immunosuppressive effects, and the current interest is in blocking the adenosine-generating enzymes or antagonize adenosine receptors for immunomodulation [99]. Bispecific T-cell engagers are a type of antibody that helps the body’s immune system destroy cancer cells by simultaneously binding to tumor antigens and T cells, forming a bridge between tumor cells and cytotoxic T cells [96]. Recently, Grover et al. reported on the promising role of F77-expressing lentiviral CAR T cells with CD28 and CD137 (4-1BB) costimulatory signals for adoptive T cell therapy of PCa [98]. Another study by Aggarwal et al. reported on the safety of, and encouraging results obtained using, a single priming dose of ^177^Lu-PSMA-617 followed by a maintenance dose of pembrolizumab in mCRPC [97]. Another phase II study by Sandhu et al. is investigating the effects of radionuclide ^177^Lu-PSMA-617 therapy versus ^177^Lu-PSMA-617 in combination with ipilimumab and nivolumab [100]. Additionally, the B7 superfamily molecule B7-H3 (CD276 or PD-L3), which is highly expressed in PCa, has also been suggested as a novel target in PCa. B7-H3 plays an immunomodulatory role and a negative regulatory role in immune response. A lower CD3+ T cell density and higher Treg density are associated with a higher B7-H3 expression, which is also correlated with androgen receptors and signaling [57]. These findings suggest B7-H3 could be a potential target for mCRPC treatment.

Further, targeting neoantigens, antiangiogenics to attenuate angiogenesis, T-cell engagers (bispecific monoclonal antibodies), and adenosine pathway inhibitors may be beneficial in turning the cold immune environment into a responsive hot environment [17]. Targeting the EIT-mediated immunosuppressive environment involving B-7-H3, B7-H4, TIM3, and LAG3, in addition to PD-1/PD-L1 and CTLA-4, may be beneficial in designing novel therapeutic strategies for PCa [18] (Figure 5). Further, targeting Tregs may lead to promising therapeutic strategies. Such strategies include the depletion of Tregs, targeting immune checkpoint inhibitors related to Tregs, tumor necrosis factor receptor engagement, and targeting Tregs-derived cytokines [19]. Targeting Tregs is also important because Tregs may be a causative factor in the failure of various treatment strategies, such as the active vaccination of cancer patients, due to the stimulation of Tregs-specific antigens.

Using multi-omics (transcriptomics, proteomics, and metabolomics) in combination with bioinformatics and machine learning may be another avenue to identify novel therapeutic targets and the cause for PCa’s resistance to treatment, which could be due to a change in gene expression. Huang et al. [101] recently reported on the C1 and C2 subtypes of PCa, with C1 showing significantly increased expression of *CGA*, *HSD17B12*, *BIRC5*, *CENPA*, and *MMP11*, as well as being associated with shorter progression-free survival. The study also concluded that BIRC5, CENPA, and MMP11 are significant therapeutic targets in PCa.

Taken together, the results of this study may be beneficial in treating mCRPC and advanced PCa. Targeting specific targets and neoantigens seems to be a promising approach because the presence of neoantigens contributes to resistance to therapy.

## 8. Completed and Ongoing Clinical Trials for PCa Therapy

Table 4 summarizes the clinical trials for mCRPC. Clinical trials including NCT00861614 (ipilimumab + radiotherapy (RT) vs. placebo + RT), NCT01057810 (ipilimumab vs. placebo), NCT03834493 (pembrolizumab + enzalutamide vs. placebo + enzalutamide), NCT03834519 (pembrolizumab + olaparib vs. next-generation hormonal agent monotherapy), NCT03834506 (pembrolizumab + docetaxel vs. docetaxel), NCT03016312 (atezolizumab + enzalutamide vs. placebo + enzalutamide), and NCT04100018 (nivolumab + docetaxel vs. placebo + docetaxel) were conducted for mCRPC but the outcome showed unimproved survival [102]. Further, B7-H3 is an attractive target in B7-H3-positive Pca, and various clinical trials are testing agents targeting it. NCT01391143 (Phase 1, completed) and NCT02923180 (Phase 2, active) tested Enoblituzumab and found a significant activation of immune response in their preliminary results. NCT03729596 (Phase 1, completed) and NCT05555117 (Phase 2, active) are being used to investigate humanized mAb vobramitamab loaded with duocarmazine targeting B7-H3, and the preliminary results showed a tolerable safety profile with a decrease in tumor lesion size in mCRPC patients. NCT04145622 is testing anti-B7-H3 ifinatamab deruxtecan (ADC DS-7300a), and NCT05914116 is investigating DB-1311, a humanized anti-B7-H3 mAb linked to a cleavable DNA topoisomerase I inhibitor for their anti-tumor activity [103]. Recently, Li et al. tested a second-generation CAR specifically targeting B7-H3 and CD28 in PCa that was positive for B7-H3 and found that they efficiently controlled the growth of the tumor and increased the release of TNF-α and IFN-γ in vitro [104].

## 9. Conclusions

The presence of an immunosuppressive tumor microenvironment, a tumor-immune microenvironment, a low tumor mutation burden, higher expression of PD-1 and low expression of PD-L1, the presence of MDSCs, tumor heterogeneity, and racial differences make prostate tumors resistant to ICIs. Although monotherapy ICI trials in mCRPC have not been very promising, combination therapies have shown some promising results. Still, there is an unmet need for a better therapeutic approach. To design novel personalized therapeutics for mCRPC, understanding the molecular mechanisms and the mechanisms contributing to resistance to ICIs in mCRPC is important. Utilizing other novel targets in the signaling pathway of PCa, along with ICIs, may be more synergistically effective. Theranostics (combining therapy with testing) is an emerging field for PCa treatment, with fewer tests. Further, PSMA therapy, a promising treatment for advanced PCa, is a type of radionuclide therapy that uses a radioactive isotope (lutetium-177) attached to a molecule that specifically binds to the prostate-specific membrane antigen (PSMA) to deliver radioactive particles directly to cancer cells. Future research activities to investigate reliable biomarkers of response to immunotherapy and the application of theranostics technology in clinical practice are warranted for an improved clinical outcome.

## Figures and Tables

**Table 1:** Anti-tumor vaccines for PCa (prepared based on the description in [39]).

Type of Vaccine	Source	Immune Response to	Against	Effects
Cell-based vaccines				
Sipuleucel-T (the only FDA-approved therapeutic cancer vaccine)	Autologous dendritic cell vaccine	Prostatic acid phosphatase (PAP) antigen	Asymptomatic or minimally symptomatic mCRPC patients with no visceral metastases	Significantly improved OS (up to 20 months)
DCVAC/PCa	Dendritic cell vaccine	Induction of immune response	mCRPC	Mixed results on OS
GVAX (irradiated whole tumor cells)	Tumor cell-based vaccine	Induce immune response to multiple TAA without the need for HLA-matching	mCRPC	Enhanced median survival
Vector-based vaccine				
PROSTVAC-VF (PSA-TRICOM)	Contain plasmid carrying transgenes coding for PSA and a triad of T cell co-stimulatory molecules LFA-3, B7.1, and ICAM-1	Elicits a strong immune response against PSA and the viral protein	Localized and advanced PCa	Inconclusive results in terms of OS or progression-free survival Better results with combination therapy
Ad5-PSA	Derived from replication-deficient recombinant adenovirus type 5	Substantial anti-PSA immune responses	mCRPC	Prolonged survival Prolonged metastasis-free survival
DNA/mRNA-Based Vaccines				
CV9103	mRNA-based vaccine encodes for PSA, PSMA, PSCA, and STEAP1	High level of cellular immunogenicity	CRPC	Safe and well-tolerated Prolonged survival

**Table 2:** Combination therapies for metastatic castration-resistant PCa (mCRPC).

Study	# of Patients	Pre-Treatment/ Therapy Strategy	Outcome
Immune Checkpoint Blockade with PARP Inhibitors			
Durvalumab plus olaparib trial [58]	17 mCRPC patients	Progression in androgen receptor blockade therapy	Median rPFS: 16.1 months PSA decline of ≥50%: 53% radiographic response: 44.4% Patients with DDR gene mutations showed better progression-free survival than those with no mutation (83.3% vs. 36.4%)
Olaparib with pembrolizumab (KEYNOTE-365, cohort A) [59]	Molecularly unselected mCRPC patients	Docetaxel-pretreated	OS of 14 months PSA response rate of 9% ORR of 8% with two partial responses Median response duration: not reached
Immune Checkpoint Blockade with Chemotherapy			
Chemotherapy plus pembrolizumab + docetaxel and prednisone [60]	104 mCRPC patients	Pembrolizumab 200 mg IV + docetaxel 75 mg/m^2^ IV Q3W and prednisone 5 mg orally twice daily	ORR-18%; median DOR of 6.7 months; PSA response rate of 28% Radiological PFS was 8.3 months OS-20.4 months
Immune Checkpoint Blockade with Radiotherapy or Cryotherapy			
Atezolizumab plus radium-223 [61]	44 mCRPC	Treatment with one 2nd generation androgen pathway inhibitor	Failed to show clinical benefit ORR: 6.8% Median radiological PFS: 3 months Median OS: 16.3 months
Ipilimumab with or without radiotherapy [62]	28 mCRPC patients were evaluated	ipilimumab alone-16 ipilimumab + radiotherapy: 34	1 patient—complete response 6 patients—stable disease 8/50 patient—>50% PSA decline
Ipilimumab against placebo following Radiotherapy CA184-043 (a phase III RCT)	799 mCRPC (1:1 randomization)	Progressed on docetaxel therapy	Median OS: 11.2 months (both groups) [63] OS was 25.2% vs. 16.6% at 2 years and 7.9% vs. 2.7% at 5 years: Ipilimumab vs. placebo [64] Median OS: 22.7 vs. 15.8 months in Ipilimumab vs placebo [63]
Pembrolizumab + cryotherapy [65]	12 newly diagnosed metastatic PC	Cryoablation with short-term androgen deprivation (8 months) and pembrolizumab (6 doses)	PFS was 14 months and PSA responses were 92%. Median systemic therapy-free survival: 17.5 months 42% of patients had PSA < 0.6 ng/ml
Immune Checkpoint Blockade with Tumor Vaccines			
Atezolizumab + sipuleucel-T [66]	37 asymptomatic or minimally symptomatic progressive mCRPC patients	Atezolizumab 1200 mg IV every 3 weeks for 2 doses SipT IV every 2 weeks	PFS: 8.2 months (atezolizumab followed by sipuleucel-T) and 5.8 months (sipuleucel-T followed by atezolizumab) Manageable safety profile
ChAdOx1-MVA 5T4 vaccine + nivolumab (ADVANCE trial) [67]	23 mCRPC patients	Two cycles of ChAdOx1-MVA 5T4 (VTP-800) vaccination and three nivolumab	22% patients—>50% reduction in PSA level Therapy well tolerated
PSA-Tricom vaccine + Ipilimumab and GM-CSF [68]	30 mCRPC patients	Chemotherapy	A decline in PSA in chemotherapy-naïve (14/24) and chemotherapy (1/6) patients Chemotherapy-naïve patients (6/14) had a median OS of 34.4 months.
CTLA-4 and PD-1/PD-L1 Combination Therapy			
Nivolumab plus ipilimumab (STARVE-PC) [69]	15 patients with mCRPC expressing AR-V7 isoform	Nivolumab 3 mg/kg plus ipilimumab 1 mg/kg every 3 weeks for four doses, then maintenance nivolumab 3 mg/kg every 2 weeks.	Promising results in patients with DDR mutations but not in others. PSA response rate (33% vs. 0%), ORR(40% vs. 0%), and OS (9.04 vs. 7.23 months) in the two subsets (DDR+ vs. DDR-) No safety concerns
Ipilimumab and nivolumab (CheckMate 650) [70]	90 mCRPC patients	45 pre-chemotherapy 45 post-chemotherapy	ORR 25% vs. 10% PSA response 17.6% vs. 10% Median OS 19.0 vs. 15.2-months Four treatment-related deaths
Durvalumab alone or durvalumab plus tremelimumab [71]	52 mCRPC patients	Previously progressed on prior abiraterone and/or enzalutamide	Durvalumab + tremelimumab: 16% vs. 0% in durvalumab alone
Tremelimumab plus durvalumab followed by durvalumab [72]	26 mCRPC patients	Tremelimumab (75 mg) plus durvalumab (1500 mg) every 4 weeks × 4 doses, followed by durvalumab (1500 mg) maintenance every 4 weeks × 9 doses	Tremelimumab plus durvalumab was safe and well tolerated. PSA declined 50% in three patients (12%). Stable disease for >6 months in six patients (24%). Median rPFS was 3.7 at a median follow-up of 43.6 months, Median overall survival was 28.1 months.
Tyrosine Kinase Inhibitors with Immune Checkpoint Blockade			
Cabozantinib with atezolizumab [73]	44 mCRPC patients in cohort 6 of the COSMIC-021 trial	Oral cabozantinib 40 mg per day and I.V. atezolizumab 1200 mg once every 3 weeks	ORR was 32% 80% disease control rate Tolerable side effects

**Table 3:** Immunotherapy of neuroendocrine and castration-resistant prostate cancer. PFS—progression-free survival; OS—overall survival; DLL3—delta-like ligand 3; mNEPC—metastatic neuroendocrine prostate cancer; CRPC—castration-resistant prostate cancer; NK cells—natural killer cells.

Neuroendocrine Prostate Cancer		
Therapeutic Agent	Patient population	Outcome
Atezolizumab [76]	Seven patients with de novo small cell or neuroendocrine tumor or transformation from preexisting adenocarcinoma	Median PFS: 3.4 months OS: 8.4 months
Pembrolizumab [77]	One patient with a refractory tumor after transformation from a preexisting adenocarcinoma after hormonal therapy-	Substantial improvement in tumor burden after 4 therapy cycles and stable disease after 21 cycles of therapy
Rova-T (DLL3 targeted antibody–drug conjugate) [78]	Rova-T was intravenously administered at a dose of 3 mg/kg to a DLL3-positive mNEPC patient every 6 weeks	A significant decrease in the size of metastatic lymph nodes with complete or partial responses in other metastatic lesion was obsereved.
YL212 (DLL3-targeted ADC)	YL212 has both extracellular and intracellular cleavage mechanisms and overcomes drug toxicity.	Clinical application is currently underway
Castration resistant prostate cancer		
Dendritic tumor cell hybridomas (aHyC) [79]	22 men with CRPC were included and 19 of them were treated with aHyC vaccine.	aHyC treatment attenuates an increase in CD56brightCD16− NK cell and benefits CRPC patient survival. Median OS was 58.5 months and cancer-specific survival was 75.7 months.

**Table 4:** Clinical trials testing the safety and efficacy of various immunotherapeutic agents for prostate cancer.

Identifier	Aim of the Study	Status	Results
NCT02082977	To investigate the safety, pharmacokinetics, pharmacodynamics, and clinical activity of EZH2 inhibitor GSK2816126	Terminated	Phase 1 clinical trial showed insufficient evidence of clinical activity of GSK2816126 [105]
NCT03480646	A study evaluating EZH2-inhibitor CPI-1205 in patients with metastatic CRPC: ProSTAR- A phase 1b/2 study	Active, not recruiting	No results posted on Clinicaltrials.gov
NCT03741712	To study the tolerance, pharmacokinetics (PK), and efficacy of EZH2 inhibitor SHR2554, alone or in combination with SHR3680, in the treatment of patients with metastatic CRPC.	Terminated	A single dose of SHR2554 50 mg was well tolerated and had a good safety profile, and its effect was significantly affected by the combined administration of itraconazole [106].
NCT03460977	A Phase 1 dose escalation and expanded cohort study of EZH2 inhibitor PF-06821497 (Mevrometostat) in CRPC. The primary aim is to confirm the safety and tolerability of PF-06821497 in combination with enzalutamide plus androgen deprivation therapy.	Recruiting patients	No results posted on Clinicaltrials.gov
NCT03093428	To study the safety and tolerability of a combination of radium-223 plus pembrolizumab, a Phase II clinical trial.	Completed	42 patients received the treatment. After 8 weeks, there was no evidence of increased CD4+ or CD8+ T-cell infiltration with treatment. However, treatment did not lead to prolonged rPFS or OS [107].
NCT05150236	To investigate the activity and safety of radionuclide 177Lu-PSMA therapy versus 177Lu-PSMA in combination with Ipilimumab and Nivolumab in patients with mCRPC- an open label, randomized, stratified, multicentre phase 2 clinical trial.	Active but not recruiting patients.	Recruiting 110 participants. No results posted on Clinicaltrials.gov
NCT05766371	A single-center, open-label study of prostate-specific membrane antigen (PSMA)-targeted radionuclide therapy with 177Lu-PSMA-617 in combination with pembrolizumab in mCRPC previously progressed on at least one prior androgen pathway inhibitor (e.g., abiraterone, enzalutamide, apalutamide).	Recruiting	A single priming dose of 177Lu-PSMA-617 followed by pembrolizumab maintenance is safe with encouraging preliminary activity in mCRPC patients [97].
NCT04446117	A Phase 3, multi-center, randomized, open-label, controlled study designed to evaluate the safety and efficacy of cabozantinib, given in combination with atezolizumab (PD-L1 inhibitor), versus a second novel hormonal therapy in men with mCRPC (CONTACT-02)	Active but not recruiting patients.	With 507 patients, cabozantinib with atezolizumab significantly improved PFS compared to second hormonal therapy in mCRPC patients with visceral metastasis [108].
NCT03007732	A non-comparative open-label multicenter Phase 2 clinical trial combining stereotactic body radiation therapy and pembrolizumab (anti-PD-1) with or without intratumoral SD-101 (TLR9 agonist) in patients with newly diagnosed hormone-naive oligometastatic prostate cancer.	Active but not recruiting patients.	TLR9 agonism, in combination with radiation and PD-1 blockade, amplifies T cell and myeloid compartments remodeling in prostate tumors and this may guide future immunotherapy strategies [109].
NCT03061539	To test the hypothesis that patients with mCRPC that have progressed following at least one line of therapy and have an immunogenic signature will respond to combined PD-1 and CTLA4 inhibition (nivolumab + ipilimumab).	Active but not recruiting patients.	Phase 2 trial results showed that nivolumab 1 mg/kg + ipilimumab 3 mg/kg had more toxicities than nivolumab 3 mg/kg + ipilimumab 1 mg/kg; the efficacy results were consistently better, suggesting the need to test a later dose schedule in a phase 3 clinical trial [110].
NCT01688492	To determine the effects of taking ipilimumab with abiraterone acetate plus prednisone in patients and prostate cancer. A phase 2 study combining ipilimumab with abiraterone acetate plus prednisone in chemotherapy and immunotherapy-naïve patients with progressive mCRPC.	Active but not recruiting patients.	No results are posted on Clinicaltrials.gov.
NCT02985957	A phase 2 trial to evaluate the effectiveness, safety, and tolerability of nivolumab followed by ipilimumab in subjects with mCRPC (CheckMate 650).	Completed	Preliminary analyses showed no clear and consistent association between efficacy and tissue or blood tumor mutational burden [70,111].
NCT03651271	An open-label, exploratory study to evaluate nivolumab with or without ipilimumab based on the percentage of tumoral CD8 cells at the time of treatment in participants with varying advanced solid tumors, including mCRPC (AMADEUS primary cohort).	Completed	Nivolumab/ipilimumab induced clinical responses and increased %CD8 in a subset of “cold” mCRPC tumors with low CD8 cells [112].
NCT05502315	A multicenter, single-arm, two-stage open-label phase 2 study of the combination of cabozantinib + nivolumab in subjects with advanced mCRPC (CANOPY).	Recruiting patients	No results are posted on Clinicaltrials.gov.
NCT05806814	Sipuleucel-T-based autologous cellular immunotherapy for advanced prostate cancer (OU-SCC-EXCITE). To evaluate the feasibility of Sipuleucel-T, given in three doses at weeks 0, 2, and 12–14, and to investigate the changes in immune response in mCRPC patients who are receiving an extended course of sipuleucel-T treatment.	Recruiting patients	No results are posted on Clinicaltrials.gov.
NCT06782555	Phase 1/2 study to test the overall safety, tolerability, and effectiveness of the combination of investigational drugs evofosfamide, zalifrelimab, and balstilimab in treating advanced or mCRPC.	Recruiting patients	No results are posted on Clinicaltrials.gov.
NCT04221542	A phase 1 study evaluating the safety, tolerability, pharmacokinetics, and efficacy of AMG 509 (xaluritamig), a STEAP1 × CD3 XmAb 2+1 immune therapy, in subjects with mCRPC.	Recruiting patients	Xaluritamig was tolerable with low-grade cytokine release syndrome (occurring primarily in cycle 1) and showed encouraging preliminary efficacy in heavily pretreated pts with mCRPC [113].
NCT06100705	An open-label, single-arm phase II study of bipolar androgen therapy, given in addition to standard-of-care Sipuleucel-T to determine the interferon gamma Enzyme-linked Immunospot (ELISPOT) response rate to PA2024 (an engineered fusion protein of prostatic acid phosphatase and granulocyte-macrophage colony-stimulating factor, which the activated autologous dendritic cells in the Sipuleucel-T vaccine are loaded with) in mCRPC patients.	Recruiting patients	No results are posted on Clinicaltrials.gov.
NCT06555796	A phase 1b, open-label, multicenter study evaluating the safety, tolerability, and efficacy of xaluritamig in subjects with high-risk biochemical recurrence of nonmetastatic castration-sensitive prostate cancer after definitive therapy	Recruiting patients	No results are posted on Clinicaltrials.gov.
NCT03866382	A phase II trial testing the effectiveness of two immunotherapy drugs (nivolumab and ipilimumab) with one anti-cancer-targeted drug (cabozantinib) for rare genitourinary tumors.	Recruiting patients	No results are posted on Clinicaltrials.gov.

## Data Availability

Not applicable.

## Abbreviations

The following abbreviations are used in this manuscript:

AR	Androgen receptor
CDK12	Cyclin-dependent kinase 12
CAR-T cells	Chimeric antigen receptor -T cell
CAFs	Cancer-associated fibroblasts
HDAC	Histone deacetylases
HMTs	Histone methyltransferase
HDMs	Histone demethylase
ICIs	Immune checkpoint inhibitors
mTOR	Mammalian target of rapamycin
mCRPC	Metastatic castration-resistant prostate cancer
MHC	Major histocompatibility complex
MDSCs	Myeloid-derived suppressor cells
PI3K	Phosphoinositide 3-kinase
PTEN	Phosphatase and tensin homolog
PD-1	Programmed cell death protein 1
PD-L1	Programmed cell death ligand 1
PSA	Prostate specific antigen
STAT3	Signal transducer and activator of transcription 3
TMB	Tumor mutational burden
TAA	Tumor-associated antigens
TILs	Tumor-infiltrating lymphocytes
TAMs	Tumor-associated macrophages
